# Retraction: Decorin inhibits glucose-induced lens epithelial cell apoptosis via suppressing p22^phox^-p38 MAPK signaling pathway

**DOI:** 10.1371/journal.pone.0358904

**Published:** 2026-09-24

**Authors:** The PLOS One Editors

After this article [1,2] was published, concerns were raised with the underlying data presented in [2]. In light of these concerns which call into question the reliability and integrity of the results in [1], the *PLOS One* Editors retract this article.

SD, JS, DX and FZ did not agree with the retraction.
